# siRNA therapy in osteoarthritis: targeting cellular pathways for advanced treatment approaches

**DOI:** 10.3389/fimmu.2024.1382689

**Published:** 2024-06-04

**Authors:** Yunshen Li, Jianan Zhao, Shicheng Guo, Dongyi He

**Affiliations:** ^1^ Department of Rheumatology, Shanghai Guanghua Hospital of Integrative Medicine, Shanghai University of Traditional Chinese Medicine, Shanghai, China; ^2^ Guanghua Clinical Medical College, Shanghai University of Traditional Chinese Medicine, Shanghai, China; ^3^ Institute of Arthritis Research in Integrative Medicine, Shanghai Academy of Traditional Chinese Medicine, Shanghai, China

**Keywords:** osteoarthritis, siRNA, chondrocytes, fibroblast-like synoviocytes, osteoblasts

## Abstract

Osteoarthritis (OA) is a common joint disorder characterized by the degeneration of cartilage and inflammation, affecting millions worldwide. The disease’s complex pathogenesis involves various cell types, such as chondrocytes, synovial cells, osteoblasts, and immune cells, contributing to the intricate interplay of factors leading to tissue degradation and pain. RNA interference (RNAi) therapy, particularly through the use of small interfering RNA (siRNA), emerges as a promising avenue for OA treatment due to its capacity for specific gene silencing. siRNA molecules can modulate post-transcriptional gene expression, targeting key pathways involved in cellular proliferation, apoptosis, senescence, autophagy, biomolecule secretion, inflammation, and bone remodeling. This review delves into the mechanisms by which siRNA targets various cell populations within the OA milieu, offering a comprehensive overview of the potential therapeutic benefits and challenges in clinical application. By summarizing the current advancements in siRNA delivery systems and therapeutic targets, we provide a solid theoretical foundation for the future development of novel siRNA-based strategies for OA diagnosis and treatment, paving the way for innovative and more effective approaches to managing this debilitating disease.

## 1 Introduction

Osteoarthritis (OA) is a prevalent chronic joint disease characterized by the degeneration of cartilage and inflammation (1). The risk factors for OA encompass obesity, injury, genetic predisposition, among others. It is noteworthy that the incidence is higher among females compared to males, and age stands as the foremost risk factor (2, 3). The pathogenesis of osteoarthritis primarily involves the articular cartilage, subchondral bone, and synovium, yet its specific mechanisms remain not fully elucidated (4). Therapeutic interventions for OA mainly include non-pharmacologic management, pharmacologic management, and surgical interventions. Recommended non-pharmacological approaches include education, self-management, exercise, and weight loss (5). Nonsteroidal anti-inflammatory drugs (NSAIDs) and acetaminophen are frontline pharmaceuticals in the treatment of OA, recommended for use in clinical guidelines. However, the cautious administration of NSAIDs is imperative to avert potential adverse effects. While acetaminophen’s efficacy falls short of that of NSAIDs, its safety profile renders it suitable for patients contraindicated for NSAIDs (6). Surgical interventions exhibit notable efficacy in long-term improvement of patients’ physiological function and alleviation of pain; nevertheless, they are not exempt from potential side effects (7). Moreover, an increasing body of evidence suggests a correlation between psychological factors and the onset of OA pain. Utilizing psychological approaches, such as coping skills training (CST) and emotional disclosure, has proven effective in alleviating OA pain (8). As the pathogenic mechanisms of OA are continually elucidated, an increasing number of pharmaceuticals targeting the pathophysiological mechanisms of the disease are under development to replace the current predominantly palliative treatments, such as chondroitin sulfate and hyaluronan, collectively known as disease-modifying osteoarthritis drugs (DMOADs). These drugs, targeting cartilage, inflammatory pathways, and subchondral bone, hold promising prospects (9).

RNA interference (RNAi) denotes the duplex RNA’s inhibitory effect on genes, constituting a natural mechanism within organisms to silence genes (10). Small interfering RNA (siRNA) represents a category of double-stranded RNA molecules, spanning 21–23 nucleotides, formed through enzymatic cleavage of double-stranded RNA (11). siRNA associates with various proteins to form the RNA-induced silencing complex (RISC). Subsequently, it pairs with target mRNA, cleaving the mRNA and ultimately impeding the transcriptional process (12). Given its selective ability to silence almost any gene, siRNA therapy holds promise as a clinical approach for targeting specific genes in the treatment of OA. The first siRNA therapy was approved in 2018, utilized for the treatment of transthyretin-mediated amyloidosis. Since then, siRNA-based therapies for various diseases have entered clinical trials, indicating the broad prospects of siRNA in treating diseases (13). Furthermore, diverse delivery systems based on siRNA have been developed to enhance the efficiency of siRNA reaching its target, encompassing polymers, lipids, antibodies, nanocarriers, and peptide segments (14). This review comprehensively summarizes the progress in utilizing siRNA for the treatment of OA from a cellular perspective, including targeting chondrocytes, fibroblast-like synoviocytes, and osteoblasts, offering a novel outlook on the application of siRNA in OA therapy.

## 2 Targeting chondrocytes

The pathological manifestations of OA are diverse, with the degeneration of articular cartilage being a primary characteristic. Articular cartilage primarily comprises water and organic extracellular components. Chondrocytes represent the exclusive cell type within the cartilage matrix, responsible for maintaining the synthesis and degradation equilibrium of the extracellular matrix (ECM) (15). Upon exposure to mechanical, inflammatory, and metabolic factors, anomalies arise in the function of chondrocytes. This results in a reduction in the generation of ECM molecules and an augmentation in the production of proteinases. The disruption of ECM homeostasis is thus initiated, consequently promoting the degeneration of articular cartilage (16).

### 2.1 Targeting the apoptosis of chondrocytes

The proliferation and apoptosis of chondrocytes undergo alterations in OA. Research indicates a diminished proliferation and an increased apoptosis of chondrocytes in OA, which is associated with the progression of OA (17–19). Targeting the proliferation and apoptosis of chondrocytes holds the potential to alleviate the progression of OA.

Downregulating the expression of DNA methyltransferase 3 alpha (*DNMT3A*) using siRNA can reduce apoptosis and induce proliferation of chondrocytes in knee OA rats (20). 15-Lipoxygenase-1 (15-LOX-1), a lipid metabolism enzyme, can facilitate apoptosis of chondrocytes induced by sodium nitroprusside (SNP) and inhibit chondrocyte proliferation. Inhibiting arachidonate 15-lipoxygenase (*ALOX15*) expression using siRNA significantly mitigates the destruction of articular surface and bone, as well as the formation of osteophytes, thereby considerably alleviating destabilization of the medial meniscus (DMM) induced OA in rats (21). The expression of KH RNA binding domain containing, signal transduction associated 1 (*KHDRBS1*) increases in chondrocytes of OA model rats stimulated by tumor necrosis factor α (TNF-α). *KHDRBS1* siRNA inhibits the activation of the nuclear factor kappa B (NF-κB) signaling pathway and significantly reduces chondrocyte apoptosis by reducing caspase-3 expression (22). Secreted phosphoprotein 1 (SPP1) is an extracellular matrix molecule that induces apoptosis of OA chondrocytes by activating the phosphatidylinositol-4,5-bisphosphate 3-kinase (PI3K)/protein kinase B (AKT) pathway and inhibits proliferation. Knocking down SPP1 using siRNA promotes chondrocyte proliferation and inhibits chondrocyte apoptosis through the reduction of caspase-3 and caspase-9 expression (23). Phosphatase and tensin homologue (*PTEN*) is a crucial tumor suppressor gene, and its expression is significantly upregulated in OA chondrocytes. *PTEN* affects cell proliferation by inhibiting the PI3K/AKT pathway. *PTEN* siRNA significantly inhibits apoptosis and promotes proliferation of OA chondrocytes (24). Downregulating ribosomal protein L38 (*RPL38*) using siRNA, leading to the upregulation of suppressor of cytokine signaling 2 (*SOCS2*) expression and activation of the janus kinase (JAK)/signal transducer and activator of transcription 3 (STAT3) pathway, resulted in a reduction of chondrocyte apoptosis induced by interleukin 1β (IL-1β). This process also alleviated histological phenomena such as reduction of articular chondrocytes, cartilage degradation and erosion in OA mice, thereby delaying the progression of OA (25).

### 2.2 Targeting chondrocyte senescence

In OA, chondrocyte senescence increases, and it can propagate to adjacent healthy chondrocytes through cell communication, inducing senescence (26). The aggregation of senescent chondrocytes promotes the progression of OA by disrupting the ECM homeostasis (27). The senescence-associated secretory phenotype (SASP) expressed by senescent chondrocytes is one of the key contributors to ECM impairment, causing an imbalance between ECM synthesis and degradation through the secretion of various cytokines and proteases such as interleukin 6 (IL-6), matrix metalloproteinases 13 (MMP13), and a disintegrin and metalloprotease with thrombospondin motifs 5 (ADAMTS5) (28). Research targeting cellular senescence provides insights for siRNA therapy.

Senescent cells reduce apoptosis by producing apoptosis inhibitor proteins (IAPs). Knocking down genes encoding anti-apoptotic proteins baculoviral IAP repeat containing 2 (*BIRC2*), baculoviral IAP repeat containing 3 (*BIRC3*), and X-linked inhibitor of apoptosis (*XIAP*) induces the clearance of senescent cells. Furthermore, the IAP inhibitor (AT-406) further alleviated cartilage degeneration and tibial subchondral bone reconstruction in rats with post-traumatic osteoarthritis (PTOA), thereby decelerating the progression of OA (29). Asporin is an extracellular matrix protein that induces chondrocyte senescence by targeting transforming growth factor β1 (TGF-β1)–Smad family member 2 (SMAD2) pathway. Knocking down asporin using siRNA inhibits senescence in chondrocytes and alleviates cartilage destruction in DMM-induced OA mice through TGF-β1 pathway (30). Inhibiting excitatory amino acid transporter protein 1 (*EAAT1*) enhances the response of senescent chondrocytes to ferroptosis, inducing cell death, with no significant impact on normal cells. Moreover, the EAAT1 inhibitor (UCPH-101) induced the clearance of senescent chondrocytes and mitigated cartilage degeneration (31). Mitofusin 2 (*MFN2*) regulates mitochondrial fusion, which plays a role in cell metabolism and aging, while dysregulation of MFN2 can lead to cartilage destruction. The expression of *MFN2* is elevated during OA and aging, while knocking down *MFN2* using siRNA can reverse age-related metabolic changes in rat chondrocytes (32). Additionally, tribbles homolog 3 (*TRB3*) siRNA reduces senescence in OA chondrocytes by reducing p16 and p21 levels, presenting another target for addressing chondrocyte senescence (33).

### 2.3 Targeting chondrocyte autophagy

The relationship between chondrocyte autophagy and apoptosis is intricate. Chondrocyte autophagy eliminates aged organelles and proteins, thereby maintaining internal homeostasis and protecting cells from apoptosis. Under the influence of external pathological factors, cellular autophagy is impaired, leading to the progression of OA (34). Hence, utilizing siRNA to restore chondrocyte autophagic function might be a means to treat OA.

The mechanistic target of rapamycin (mTOR) pathway is a crucial cellular autophagy inhibition pathway, regulated by PI3K/AKT and AMP-activated protein kinase (AMPK) (35). MicroRNA 7 (*miR-7*) siRNA inhibits the phosphorylation of the PI3K/AKT/mTOR pathway by reducing interleukin 17A (*IL-17A*) expression, and promotes the conversion of microtubule associated protein light chain 3 (LC3) from LC3-I to LC3-II, enhances beclin 1 (*BECN1*) expression, and suppresses sequestosome 1 (*SQSTM1*) expression, thereby restoring autophagic dysfunction in IL-1β induced chondrocytes. This significantly reduces cartilage destruction and the progression of OA in model rats (36). The upregulation of *MFN2* in OA model mice, through activating the NF-κB and p38 mitogen-activated protein kinase (MAPK) pathways and inhibiting the PI3K/AKT/mTOR pathway, promotes inflammation and leads to excessive autophagy in chondrocytes. Knocking down *MFN2* can suppress inflammation and cartilage degeneration in OA rat chondrocytes, thereby slowing down the progression of OA (37). The upregulation of transient receptor potential cation channel subfamily V member 5 (*TRPV5*) expression in OA is mitigated by *TRPV5* siRNA, which reduces intracellular Ca^2+^ influx and enhances autophagy in MIA-induced OA rat chondrocytes. Furthermore, ruthenium red (a TRPV5 inhibitor) delays OA progression by reducing cartilage destruction (38). TRB3 inhibits autophagy by suppressing the autophagic receptor p62 and is upregulated in TNF-α-induced OA chondrocytes. Knocking down *TRB3* using siRNA promotes autophagy in chondrocytes, making it a potential target for targeting chondrocyte autophagic function (33).

### 2.4 Targeting chondrocyte secretion

In OA, the aberrant function of chondrocytes leads to the release of degradative enzymes, such as matrix metalloproteinases (MMPs) and a disintegrin and metalloprotease with thrombospondin motifs (ADAMTSs) (4). These enzymes further contribute to cartilage degradation. The degradation products of cartilage can serve as damage-associated molecular patterns (DAMPs), entering the synovium and inducing the production of inflammatory factors. This, in turn, further stimulates chondrocytes to produce degradative enzymes, forming a vicious cycle (39).

*MMP13* is upregulated in PTOA and contributes to cartilage destruction by degrading type II collagen. Using a nano-platform to deliver *MMP13* siRNA reduces cartilage degradation, synovial hyperplasia and osteophyte growth in PTOA mice model, slowing down the progression of PTOA with favorable long-term therapeutic effects (40). Intra-articular injection of *MMP13* siRNA and/or *ADAMTS5* siRNA inhibited cartilage degradation in early-stage OA mice model (41). The use of nanoparticle delivery for lysine demethylase 6B (*KDM6B*) siRNA lowered the expression of *MMP13* in mice model, and significantly alleviated the progression of OA by reducing cartilage degradation (42). The level of β-catenin is increased in chondrocytes of OA mice. Catenin beta 1 (*CTNNB1*) encodes β-catenin, and knocking down *CTNNB1* reduces the expression of matrix metalloproteinases 3 (*MMP3*), *MMP13*, and a disintegrin and metalloprotease with thrombospondin motifs 4 (*ADAMTS4*). This suggests that *CTNNB1* siRNA may inhibit cartilage degradation (43). Jian Zhang et al. discovered a drug that co-delivers curcumin and endothelial PAS domain protein 1 (*EPAS1*) siRNA, which alleviates mice cartilage degradation and slows down the progression of OA by reducing the expression of *MMP3*, *MMP13*, *ADAMTS5* in OA chondrocytes (44). The activation of the interleukin 1 (IL-1) signaling pathway can lead to the progression of OA. Matrix metalloproteinases 9 (*MMP9*) siRNA reduces the shedding of syndecan-4 (SDC4), thereby lowering the sensitivity of chondrocytes to the IL-1 signaling pathway (45). Furthermore, *miR-7* siRNA inhibits the expression of *MMP3*, *MMP13*, and *ADAMTS5* in IL-1β-induced chondrocytes, thereby promoting ECM homeostasis and delaying OA progression (36).

## 3 Targeting fibroblast-like synoviocytes

Synovial inflammation is another characteristic feature of OA. The aberrant function of chondrocytes leads to the secretion of proteolytic enzymes, causing the production of inflammatory and metabolic products that affect the adjacent synovium. The progression of synovial inflammation can further exacerbate cartilage damage (46).

The production of pro-inflammatory cytokines such as IL-1β, TNF-α, IL-6, can exacerbate cartilage degradation and is associated with hyperalgia in OA (47, 48). Silencing NLR family pyrin domain containing 1 (*NLRP1*) reduces the production of IL-1β induced by P2X4 purinoceptor (P2X4) in OA fibroblast-like synoviocytes (49). RELA proto-oncogene, NF-kB subunit (*RELA*) siRNA significantly inhibits the induction of IL-1β and TNF-α in the synovial fluid, thereby alleviating synovial inflammation and cartilage degradation in early-stage OA rats through inhibition of the NF-κB pathway (50). Utilizing nanoparticles to deliver cyclin dependent kinase inhibitor 2A (*CDKN2A*) siRNA results in a reduction of *IL-1β*, *IL-6*, and *TNF-α* expression in fibroblast-like synoviocytes of model rats, and reduces cartilage destruction and pain (51). Hyaluronan contributes to the composition of synovial fluid for joint lubrication. In OA, hyaluronan degradation occurs, promoting inflammation. The expression of cell migration inducing hyaluronidase 1 (*CEMIP*) increases in OA fibroblast-like synoviocytes, and knocking down *CEMIP* reduces the degradation of hyaluronan in OA fibroblast-like synoviocytes (52). Glutaminase (*GLS*) siRNA inhibits glutamine–glutamate metabolism, reducing the IL-6 inflammatory response in OA fibroblast-like synoviocytes (53).

Proliferation of fibroblast-like synoviocytes is one of the characteristics of synovial inflammation and can lead to hyperplasia of the synovial lining (54). Knocking down latent transforming growth factor beta binding protein 1 (*LTBP-1*) using siRNA reduces fibroblast-like synoviocytes proliferation by downregulating the transforming growth factor β (TGF-β) signaling pathway (55).

Methyltransferase like 3 (METTL3) mediated m6A modification inhibits autophagy in OA fibroblast-like synoviocytes by regulating autophagy related 7 (*ATG7*) RNA and promotes cell senescence. *METTL3* siRNA is able to inhibit expression of SASP-related genes and alleviate senescence in fibroblast-like synoviocytes. Furthermore, intra-articular injection of METTL3 siRNA delayed the progression of DMM-induced OA in mice (56). Fibroblast-like synoviocytes are considered the primary cells involved in synovial fibrosis in OA, and chronic joint pain is closely associated with synovial fibrosis (57). Inhibiting the upregulated expression of hypoxia inducible factor 1 subunit alpha (*HIF-1A*) in knee OA model rats reduces pyroptosis in fibroblast-like synoviocytes and significantly decreases the expression of synovial fibrogenic markers (58).

## 4 Targeting osteoblasts

In OA, subchondral bone undergoes remodeling due to excessive loading and changes in mechanical environment (59). The abnormal remodeling of subchondral bone can further exacerbate cartilage degradation (60). Osteoblasts regulate bone formation, remodeling and mineralization (61). Osteoblasts undergo changes in phenotype in OA, such as elevated alkaline phosphatase activity and increased secretion of osteocalcin. Additionally, alterations in signaling pathways like wingless-type MMTV integration site family (WNT) and TGF-β contribute to the abnormal remodeling of subchondral bone (62, 63). Hence, targeting osteoblasts with siRNA represents a potential therapeutic approach.

In OA, there is an increased production of endogenous hepatocyte growth factor (HGF), which, by stimulating the production of TGF-β1, inhibits osteoblast responsiveness to bone morphogenetic protein 2 (BMP-2), leading to abnormal mineralization. *HGF* siRNA restores osteoblast responsiveness to BMP-2 and upregulates the WNT signaling pathway to nearly normal levels (64). Leptin expression is significantly increased in OA osteoblasts and can lead to elevated levels of alkaline phosphatase and osteocalcin, as well as increased osteoblast proliferation. Partially reducing the alkaline phosphatase activity and osteocalcin release is possible by inhibiting leptin or its receptor using siRNA, indicating the potential to inhibit bone remodeling (65). Silencing *ALOX15* promotes AMPK phosphorylation, inhibits mechanistic target of rapamycin complex 1 (mTORC1) phosphorylation, thereby suppressing expression levels of *TGF-β1*. This, in turn, enhances osteoblast autophagy and ultimately alleviates the progression of OA (66). Activation of Toll-like receptor 4 (TLR4) and innate immune activation can exacerbate cartilage degradation in OA. The antidepressant amitriptyline can bind to TLR4, inhibiting TLR4, IL-1 receptor, and NLR family pyrin domain containing 3 (NLRP3) dependent innate immune responses in OA chondrocytes, synoviocytes, and osteoblasts. Similarly, silencing *NLRP3* using siRNA has a comparable effect (67).

## 5 Targeting other cells

The pathological changes in OA involve the entire joint, and current research predominantly focuses on the abnormalities in cartilage. Research on other cells to treat OA is limited, such as macrophages or mesenchymal stem cells (68). Nevertheless, siRNA targeting these cells has shown promising prospects.

Xu Chen et al. engineered nanoparticles capable of releasing nitric oxide and notch receptor 1 (*NOTCH1*) siRNA. By inhibiting macrophage inflammatory responses, these nanoparticles reduced OA cartilage damage without significant side effects (69).

Mesenchymal stem cells derived from bone marrow are ideal for tissue repair due to their differentiation potential. Inhibiting BLACAT1 overlapping LEMD1 locus (*BLACAT1*) promotes the proliferation and osteogenic differentiation of bone marrow mesenchymal stem cells under inflammatory conditions, demonstrating potential for treating OA (70).

## 6 Discussion

OA is a chronic degenerative joint disease that, as it progresses, often leads to disability and pain, significantly impacting the quality of life (71). Currently, the treatment approach for OA primarily focuses on mitigating disease symptoms and arresting its progression. In recent years, with an increasingly profound understanding of OA, it is recognized as a comprehensive, multifactorial joint disorder, intimately connected to the interactions among articular cartilage, synovium, subchondral bone, and their constituent cells. Due to its inherent property of selectively silencing genes, siRNA stands as a potential candidate among DMOADs. Recent investigations have disclosed that employing siRNA to target chondrocytes, fibroblast-like synoviocytes, osteoblasts, osteoblasts, and other cells and molecules involved in the development of OA can effectively decelerate the progression of the ailment. Additionally, a substantial volume of research centered around siRNA delivery systems has enhanced the precision and duration of siRNA delivery to target tissues. While there is currently no siRNA therapy specifically targeting osteoarthritis (according to clinicaltrials.gov), an increasing number of siRNA therapies targeting various diseases are entering clinical trials or gaining approval. These include cancer, hypertension, hypercholesterolemia, as well as some rare genetic disorders such as hemophilia and primary hyperoxaluria (72). These studies collectively highlight the broad potential of employing siRNA in the treatment of OA.

However, the constraints of current siRNA therapies cannot be disregarded. The primary method of siRNA therapy involves intravenous administration using nano-carriers, including lipid carriers, polymer carriers, and inorganic carriers (12). Lipid carriers, due to their positively charged nature, may undergo aggregation with serum proteins (73). On the other hand, negatively charged siRNA can distribute through the bloodstream to the reticuloendothelial system (RES) and are more readily phagocytosed, compared to neutral or positively charged counterparts (11). Furthermore, siRNA permits a degree of mismatch with the target mRNA, resulting in the silencing of non-target genes, known as off-target effects (74). Competition with the endogenous RNAi pathway and off-target effects can lead to hepatotoxicity (75). Cationic lipids and polymers, among other delivery methods involving internalization, can also induce immune responses by activating Toll-like receptor 7 (TLR7) and Toll-like receptor 8 (TLR8) (76). To address these issues, numerous improvement strategies have been applied to optimize siRNA delivery, including chemical modifications, siRNA-ligand conjugation, siRNA-polymer conjugation, and others (77).

Beyond the inherent limitations of siRNA therapy, the selection of target genes represents one of the challenges in utilizing siRNA therapy for OA treatment. Given that OA is a disease affecting the entire joint, silencing target genes for OA may also potentially diminish therapeutic efficacy through the impact on physiological activities of other tissues, or even inadvertently accelerate disease progression. The activation of the PI3K/AKT/mTOR pathway can enhance skeletal muscle protein synthesis, whereas inhibition of the PI3K/AKT/mTOR pathway can increase protein breakdown, marking a signature of muscle atrophy. Moreover, muscular weakness constitutes a risk factor in the development of OA (78, 79). IL-6 is released during and after exercise to enhance muscle energy supply and plays a crucial role in the repair of acute muscle injuries by activating satellite cells (80, 81). Inhibiting IL-6 could have repercussions on muscle function, potentially exacerbating the progression of OA. 

**Figure 1:**
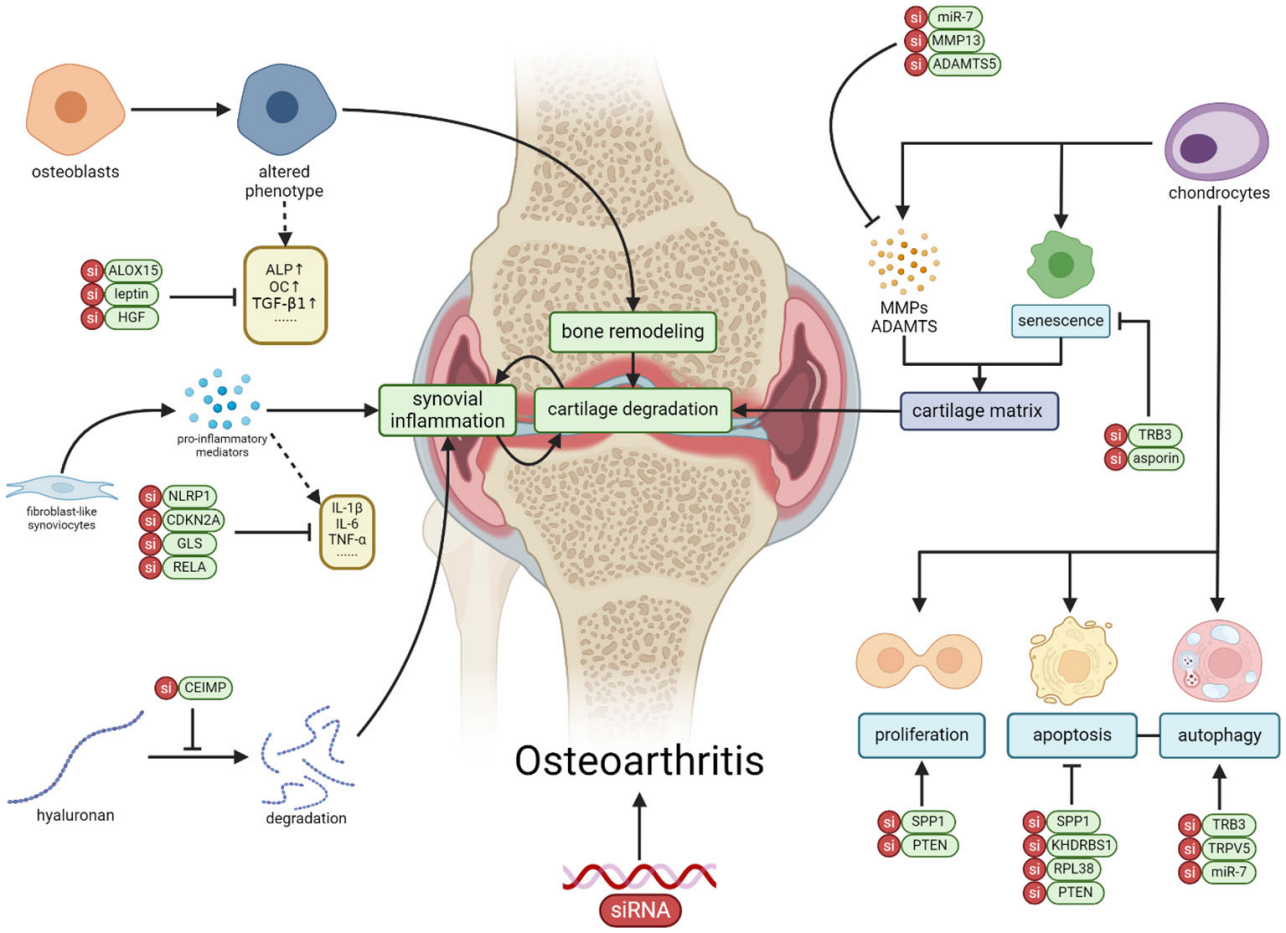
The application of siRNA therapy in OA involves targeting genes and signaling pathways in various cell types within OA, including chondrocytes, synovial fibroblasts, osteoblasts, macrophages, and mesenchymal stem cells. Through the correction of cellular activities such as proliferation, apoptosis, senescence, autophagy, and inflammation, siRNA exhibits the potential to alleviate the progression of OA (Created with BioRender.com).

In this review, we summarized the advancements in utilizing siRNA to target various cells for the treatment of OA, including chondrocytes, fibroblast-like synoviocytes, osteoblasts, and others (**Table 1** and **Figure 1**). These studies, encompassing both *in vitro* experiments and *in vivo* trials, consistently demonstrate the potential of siRNA therapy. However, siRNA therapy still confronts numerous challenges, such as the selection of therapeutic targets, more efficient carriers for siRNA delivery, and addressing the immunogenicity of siRNA. Hence, the therapeutic potential of siRNA remains vast, with significant room for further advancement. 

**Table 1:** Gene targets, cell activity and pathways table.

Cell	Cell Activity	Gene	Pathway	Reference
Chondrocytes	Proliferation	DNMT3A,ALOX15,KHDRBS1, SPP1,PTEN,RPL38	NF-κB PI3K/AKT/mTOR JAK/STAT3 TGF-β1–SMAD2 p38 MAPK IL-1	(20–25, 29, 30) (31–33, 36–38, 40–45)
	Senescence	BIRC2,BIRC3,XIAP,asporin,EAAT1,MFN2,TRB3		
	Autophagy	miR-7,MFN2,TRPV5,TRB3		
	Secretion	MMP13,ADAMTS5,KDM6B,CTNNB1,EPAS1,MMP9,miR-7		
Fibroblast-like synoviocytes	Inflammation	NLRP1,RELA,CDKN2A,CEMIP,GLS,LTBP-1	NF-κB TGF-β	(49, 50–55, 56, 58)
	Senescence and pyroptosis	METTL3,HIF-1A		
Osteoblasts	Bone remodeling	HGF,leptin,ALOX15,NLRP3	WNT AMPK	(64–67)

## Author contributions

YL: Writing – original draft, Writing – review & editing. JZ: Writing – original draft, Writing – review & editing. SG: Writing – review & editing. DH: Writing – review & editing.

## Glossary

**Table:**

DNMT3A	DNA methyltransferase 3 alpha
ALOX15	Arachidonate 15-lipoxygenase
KHDRBS1	KH RNA binding domain containing, signal transduction associated 1
TNF-α	Tumor necrosis factor α
NF-κB	Nuclear factor kappa B
SPP1	Secreted phosphoprotein 1
PI3K	Phosphatidylinositol-4,5-bisphosphate 3-kinase
AKT	Protein kinase B
PTEN	Phosphatase and tensin homologue
RPL38	Ribosomal protein L38
SOCS2	Suppressor of cytokine signaling 2
JAK	Janus kinase
STAT3	Signal transducer and activator of transcription 3
IL-1β	Interleukin 1β
IL-6	Interleukin 6
MMP13	Matrix metalloproteinases 13
ADAMTS5	A disintegrin and metalloprotease with thrombospondin motifs 5
BIRC2	Baculoviral IAP repeat containing 2
BIRC3	Baculoviral IAP repeat containing 3
XIAP	X-linked inhibitor of apoptosis
TGF-β1	Transforming growth factor β1
Smad2	Smad family member 2
EAAT1	Excitatory amino acid transporter protein 1
MFN2	Mitofusin 2
TRB3	Tribbles homolog 3
mTOR	Mechanistic target of rapamycin
AMPK	AMP-activated protein kinase
miR-7	MicroRNA 7
IL-17A	Interleukin 17A
BECN1	Beclin 1
SQSTM1	Sequestosome 1
p38 MAPK	p38 mitogen-activated protein kinase
TRPV5	Transient receptor potential cation channel subfamily V member 5
KDM6B	Lysine demethylase 6B
CTNNB1	Catenin beta 1
MMP3	Matrix metalloproteinases 3
ADAMTS4	A disintegrin and metalloprotease with thrombospondin motifs 4
EPAS1	endothelial PAS domain protein 1
IL-1	Interleukin 1
MMP9	Matrix metalloproteinases 9
NLRP1	NLR family pyrin domain containing 1
RELA	RELA proto-oncogene, NF-kB subunit
CDKN2A	Cyclin dependent kinase inhibitor 2A
CEMIP	Cell migration inducing hyaluronidase 1
GLS	Glutaminase
LTBP-1	Latent transforming growth factor beta binding protein 1
TGF-β	Transforming growth factor β
METTL3	Methyltransferase like 3
ATG7	Autophagy related 7
HIF-1A	Hypoxia inducible factor 1 subunit alpha
WNT	Wingless-type MMTV integration site family
HGF	Hepatocyte growth factor
BMP-2	Bone morphogenetic protein 2
mTORC1	Mechanistic target of rapamycin complex 1
TLR4	Toll-like receptor 4
NLRP3	NLR family pyrin domain containing 3
NOTCH1	Notch receptor 1
BLACAT1	BLACAT1 overlapping LEMD1 locus

## References

[1] Molnar V Matišić V Kodvanj I Bjelica R Jeleč Ž Hudetz D. Cytokines and chemokines involved in osteoarthritis pathogenesis. Int J Mol Sci. (2021) 22:9208. doi: 10.3390/ijms22179208 34502117 PMC8431625

[2] Vina ER Kwoh CK. Epidemiology of osteoarthritis: literature update. Curr Opin Rheumatol. (2018) 30:160–7. doi: 10.1097/BOR.0000000000000479 PMC5832048 29227353

[3] Hunter DJ Bierma-Zeinstra S. Osteoarthritis. Lancet. (2019) 393:1745–59. doi: 10.1016/S0140-6736(19)30417-9 31034380

[4] Glyn-Jones S Palmer AJR Agricola R Price AJ Vincent TL Weinans H. Osteoarthritis. Lancet. (2015) 386:376–87. doi: 10.1016/S0140-6736(14)60802-3 25748615

[5] Nelson AE Allen KD Golightly YM Goode AP Jordan JM. A systematic review of recommendations and guidelines for the management of osteoarthritis: The Chronic Osteoarthritis Management Initiative of the U.S. Bone and Joint Initiative. Semin Arthritis Rheumatol. (2014) 43:701–12. doi: 10.1016/j.semarthrit.2013.11.012 24387819

[6] Abramoff B Caldera FE. Osteoarthritis: pathology, diagnosis, and treatment options. Med Clin North Am. (2020) 104:293–311. doi: 10.1016/j.mcna.2019.10.007 32035570

[7] de l’Escalopier N Anract P Biau D. Surgical treatments for osteoarthritis. Ann Phys Rehabil Med. (2016) 59:227–33. doi: 10.1016/j.rehab.2016.04.003 27185463

[8] Keefe FJ Somers TJ. Psychological approaches to understanding and treating arthritis pain. Nat Rev Rheumatol. (2010) 6:210–6. doi: 10.1038/nrrheum.2010.22 20357790

[9] Huang Z Ding C Li T Yu SPC. Current status and future prospects for disease modification in osteoarthritis. Rheumatology. (2018) 57:iv108–23. doi: 10.1093/rheumatology/kex496 29272498

[10] Downward J. RNA interference. BMJ. (2004) 328:1245–8. doi: 10.1136/bmj.328.7450.1245 PMC416605 15155505

[11] Alshaer W Zureigat H Al Karaki A Al-Kadash A Gharaibeh L Hatmal MM. siRNA: Mechanism of action, challenges, and therapeutic approaches. Eur J Pharmacol. (2021) 905:174178. doi: 10.1016/j.ejphar.2021.174178 34044011

[12] Friedrich M Aigner A. Therapeutic siRNA: state-of-the-art and future perspectives. BioDrugs Clin Immunother Biopharm Gene Ther. (2022) 36:549–71. doi: 10.1007/s40259-022-00549-3 PMC9396607 35997897

[13] Dong Y Siegwart DJ Anderson DG. Strategies, design, and chemistry in siRNA delivery systems. Adv Drug Delivery Rev. (2019) 144:133–47. doi: 10.1016/j.addr.2019.05.004 PMC6745264 31102606

[14] Kanasty R Dorkin JR Vegas A Anderson D. Delivery materials for siRNA therapeutics. Nat Mater. (2013) 12:967–77. doi: 10.1038/nmat3765 24150415

[15] Martel-Pelletier J Barr AJ Cicuttini FM Conaghan PG Cooper C Goldring MB. Osteoarthritis. Nat Rev Dis Primer. (2016) 2:16072. doi: 10.1038/nrdp.2016.72 27734845

[16] Li MH Xiao R Li JB Zhu Q. Regenerative approaches for cartilage repair in the treatment of osteoarthritis. Osteoarthritis Cartilage. (2017) 25:1577–87. doi: 10.1016/j.joca.2017.07.004 28705606

[17] Zou L Liu J Lu H. Influence of protein kinase RIPK4 expression on the apoptosis and proliferation of chondrocytes in osteoarthritis. Mol Med Rep. (2018) 17:3078–84. doi: 10.3892/mmr.2017.8209 PMC5783529 29257245

[18] Zhu Z Gao S Chen C Xu W Xiao P Chen Z. The natural product salicin alleviates osteoarthritis progression by binding to IRE1α and inhibiting endoplasmic reticulum stress through the IRE1α-IκBα-p65 signaling pathway. Exp Mol Med. (2022) 54:1927–39. doi: 10.1038/s12276-022-00879-w PMC9722708 36357568

[19] Tian F Wang J Zhang Z Yang J. LncRNA SNHG7/miR-34a-5p/SYVN1 axis plays a vital role in proliferation, apoptosis and autophagy in osteoarthritis. Biol Res. (2020) 53:9. doi: 10.1186/s40659-020-00275-6 32066502 PMC7027214

[20] Gu W Shi Z Song G Zhang H. MicroRNA-199–3p up-regulation enhances chondrocyte proliferation and inhibits apoptosis in knee osteoarthritis via DNMT3A repression. Inflammation Res. (2021) 70:171–82. doi: 10.1007/s00011-020-01430-1 33433641

[21] Chen K Yan Y Li C Yuan J Wang F Huang P. Increased 15-lipoxygenase-1 expression in chondrocytes contributes to the pathogenesis of osteoarthritis. Cell Death Dis. (2017) 8:e3109. doi: 10.1038/cddis.2017.511 29022900 PMC5682676

[22] Xu L Sun C Zhang S Xu X Zhai L Wang Y. Sam68 promotes NF-κB activation and apoptosis signaling in articular chondrocytes during osteoarthritis. Inflammation Res. (2015) 64:895–902. doi: 10.1007/s00011-015-0872-3 26350037

[23] Lin Z Tian XY Huang XX He LL Xu F. microRNA-186 inhibition of PI3K-AKT pathway via SPP1 inhibits chondrocyte apoptosis in mice with osteoarthritis. J Cell Physiol. (2019) 234:6042–53. doi: 10.1002/jcp.27225 30500068

[24] Huang Z Zhang N Ma W Dai X Liu J. MiR-337–3p promotes chondrocytes proliferation and inhibits apoptosis by regulating PTEN/AKT axis in osteoarthritis. BioMed Pharmacother. (2017) 95:1194–200. doi: 10.1016/j.biopha.2017.09.016 28931211

[25] Shi L Hu H Sun P Li Z Ji L Liu S. RPL38 knockdown inhibits the inflammation and apoptosis in chondrocytes through regulating METTL3-mediated SOCS2 m6A modification in osteoarthritis. Inflammation Res. (2022) 71:977–89. doi: 10.1007/s00011-022-01579-x 35596790

[26] Xie J Wang Y Lu L Liu L Yu X Pei F. Cellular senescence in knee osteoarthritis: molecular mechanisms and therapeutic implications. Ageing Res Rev. (2021) 70:101413. doi: 10.1016/j.arr.2021.101413 34298194

[27] Loeser RF. Aging and osteoarthritis: the role of chondrocyte senescence and aging changes in the cartilage matrix. Osteoarthritis Cartilage. (2009) 17:971–9. doi: 10.1016/j.joca.2009.03.002 PMC2713363 19303469

[28] Yagi M Endo K Komori K Sekiya I. Comparison of the effects of oxidative and inflammatory stresses on rat chondrocyte senescence. Sci Rep. (2023) 13:7697. doi: 10.1038/s41598-023-34825-1 37169906 PMC10175275

[29] Peilin W Songsong T Chengyu Z Zhi C Chunhui M Yinxian Y. Directed elimination of senescent cells attenuates development of osteoarthritis by inhibition of c-IAP and XIAP. Biochim Biophys Acta Mol Basis Dis. (2019) 1865:2618–32. doi: 10.1016/j.bbadis.2019.05.017 31251987

[30] Liu L Zhao C Zhang H Lu Y Luo B Yao Z. Asporin regulated by miR-26b-5p mediates chondrocyte senescence and exacerbates osteoarthritis progression via TGF-β1/Smad2 pathway. Rheumatol Oxf. (2022) 61:2631–43. doi: 10.1093/rheumatology/keab725 34559207

[31] Wen Z Xia G Liang C Wang X Huang J Zhang L. Selective clearance of senescent chondrocytes in osteoarthritis by targeting excitatory amino acid transporter protein 1 to induce ferroptosis. Antioxid Redox Signal. (2023) 39:262–77. doi: 10.1089/ars.2022.0141 36601724

[32] Xu L Wu Z He Y Chen Z Xu K Yu W. MFN2 contributes to metabolic disorders and inflammation in the aging of rat chondrocytes and osteoarthritis. Osteoarthritis Cartilage. (2020) 28:1079–91. doi: 10.1016/j.joca.2019.11.011 32416221

[33] Gu Y Yan R Wang Y Zeng Y Yao Q. High TRB3 expression induces chondrocyte autophagy and senescence in osteoarthritis cartilage. Aging. (2022) 14:5366–75. doi: 10.18632/aging.204066 PMC9320551 35776529

[34] Xu K He Y Moqbel SAA Zhou X Wu L Bao J. SIRT3 ameliorates osteoarthritis via regulating chondrocyte autophagy and apoptosis through the PI3K/Akt/mTOR pathway. Int J Biol Macromol. (2021) 175:351–60. doi: 10.1016/j.ijbiomac.2021.02.029 33556400

[35] Dalle Pezze P Ruf S Sonntag AG Langelaar-Makkinje M Hall P. A systems study reveals concurrent activation of AMPK and mTOR by amino acids. Nat Commun. (2016) 7:13254. doi: 10.1038/ncomms13254 27869123 PMC5121333

[36] Zhou X Li J Zhou Y Yang Z Yang H Li D. Down-regulated ciRS-7/up-regulated miR-7 axis aggravated cartilage degradation and autophagy defection by PI3K/AKT/mTOR activation mediated by IL-17A in osteoarthritis. Aging. (2020) 12:20163–83. doi: 10.18632/aging.103731 PMC7655186 33099538

[37] Deng X Xu H Pan C Hao X Liu J Shang X. Moderate mechanical strain and exercise reduce inflammation and excessive autophagy in osteoarthritis by downregulating mitofusin 2. Life Sci. (2023) 332:122020. doi: 10.1016/j.lfs.2023.122020 37579836

[38] Wei Y Wang Y Wang Y Bai L. Transient receptor potential vanilloid 5 mediates ca2+ Influx and inhibits chondrocyte autophagy in a rat osteoarthritis model. Cell Physiol Biochem. (2017) 42:319–32. doi: 10.1159/000477387 28535500

[39] Lambert C Zappia J Sanchez C Florin A Dubuc JE Henrotin Y. The damage-associated molecular patterns (DAMPs) as potential targets to treat osteoarthritis: perspectives from a review of the literature. Front Med. (2020) 7:607186. doi: 10.3389/fmed.2020.607186 PMC7847938 33537330

[40] Bedingfield SK Colazo JM Di Francesco M Yu F Liu DD Di Francesco V. Top-Down Fabricated microPlates for Prolonged, Intra-articular Matrix Metalloproteinase 13 siRNA Nanocarrier Delivery to Reduce Post-traumatic Osteoarthritis. ACS Nano. (2021) 15:14475–91. doi: 10.1021/acsnano.1c04005 PMC9074946 34409835

[41] Hoshi H Akagi R Yamaguchi S Muramatsu Y Akatsu Y Yamamoto Y. Effect of inhibiting MMP13 and ADAMTS5 by intra-articular injection of small interfering RNA in a surgically induced osteoarthritis model of mice. Cell Tissue Res. (2017) 368:379–87. doi: 10.1007/s00441-016-2563-y 28120109

[42] Jin Y Liu Z Li Z Li H Zhu C Li R. Histone demethylase JMJD3 downregulation protects against aberrant force-induced osteoarthritis through epigenetic control of NR4A1. Int J Oral Sci. (2022) 14:34. doi: 10.1038/s41368-022-00190-4 35831280 PMC9279410

[43] Hu S Mao G Zhang Z Wu P Wen X Liao W. MicroRNA-320c inhibits development of osteoarthritis through downregulation of canonical Wnt signaling pathway. Life Sci. (2019) 228:242–50. doi: 10.1016/j.lfs.2019.05.011 31075235

[44] Zhang ZJ Hou YK Chen MW Yu XZ Chen SY Yue YR. A pH-responsive metal-organic framework for the co-delivery of HIF-2α siRNA and curcumin for enhanced therapy of osteoarthritis. J Nanobiotechnol. (2023) 21:18. doi: 10.1186/s12951-022-01758-2 PMC9847079 36650517

[45] Bollmann M Pinno K Ehnold LI Märtens N Märtson A Pap T. MMP-9 mediated Syndecan-4 shedding correlates with osteoarthritis severity. Osteoarthritis Cartilage. (2021) 29:280–9. doi: 10.1016/j.joca.2020.10.009 33246160

[46] Berenbaum F. Osteoarthritis as an inflammatory disease (osteoarthritis is not osteoarthrosis)! Osteoarthritis Cartilage. (2013) 21:16–21. doi: 10.1016/j.joca.2012.11.012 23194896

[47] Liao Y Ren Y Luo X Mirando AJ Long JT Leinroth A. Interleukin-6 signaling mediates cartilage degradation and pain in posttraumatic osteoarthritis in a sex-specific manner. Sci Signal. (2022) 15:eabn7082. doi: 10.1126/scisignal.abn7082 35881692 PMC9382892

[48] Sellam J Berenbaum F. The role of synovitis in pathophysiology and clinical symptoms of osteoarthritis. Nat Rev Rheumatol. (2010) 6:625–35. doi: 10.1038/nrrheum.2010.159 20924410

[49] Fan C Zhao X Guo X Cao X Cai J. P2X4 promotes interleukin−1β production in osteoarthritis via NLRP1. Mol Med Rep. (2014) 9:340–4. doi: 10.3892/mmr.2013.1748 24145861

[50] Chen LX Lin L Wang HJ Wei XL Fu X Zhang JY. Suppression of early experimental osteoarthritis by in *vivo* delivery of the adenoviral vector-mediated NF-kappaBp65-specific siRNA. Osteoarthritis Cartilage. (2008) 16:174–84. doi: 10.1016/j.joca.2007.06.006 17686636

[51] Park H Lee HR Shin HJ Park JA Joo Y Kim SM. p16INK4a-siRNA nanoparticles attenuate cartilage degeneration in osteoarthritis by inhibiting inflammation in fibroblast-like synoviocytes. Biomater Sci. (2022) 10:3223–35. doi: 10.1039/D1BM01941D 35579255

[52] Shiozawa J de Vega S Cilek MZ Yoshinaga C Nakamura T Kasamatsu S. Implication of HYBID (Hyaluronan-binding protein involved in hyaluronan depolymerization) in hyaluronan degradation by synovial fibroblasts in patients with knee osteoarthritis. Am J Pathol. (2020) 190:1046–58. doi: 10.1016/j.ajpath.2020.01.003 32084364

[53] Farah H Wijesinghe SN Nicholson T Alnajjar F Certo M Alghamdi A. Differential metabotypes in synovial fibroblasts and synovial fluid in hip osteoarthritis patients support inflammatory responses. Int J Mol Sci. (2022) 23. doi: 10.3390/ijms23063266 PMC8950319 35328687

[54] Sanchez-Lopez E Coras R Torres A Lane NE Guma M. Synovial inflammation in osteoarthritis progression. Nat Rev Rheumatol. (2022) 18:258–75. doi: 10.1038/s41584-022-00749-9 PMC9050956 35165404

[55] Wang X Dong C Li N Ma Q Yun Z Cai C. Modulation of TGF−β activity by latent TGF−β−binding protein 1 in human osteoarthritis fibroblast−like synoviocytes. Mol Med Rep. (2018) 17:1893–900. doi: 10.3892/mmr.2017.8086 29257223

[56] Chen X Gong W Shao X Shi T Zhang L Dong J. METTL3-mediated m(6)A modification of ATG7 regulates autophagy-GATA4 axis to promote cellular senescence and osteoarthritis progression. Ann Rheum Dis. (2022) 81:87–99. doi: 10.1136/annrheumdis-2021-221091 34706873

[57] Maglaviceanu A Wu B Kapoor M. Fibroblast-like synoviocytes: Role in synovial fibrosis associated with osteoarthritis. Wound Repair Regen. (2021) 29:642–9. doi: 10.1111/wrr.12939 34021514

[58] Zhang L Zhang L Huang Z Xing R Li X Yin S. Increased HIF-1α in knee osteoarthritis aggravate synovial fibrosis via fibroblast-like synoviocyte pyroptosis. Oxid Med Cell Longev. (2019) 2019:6326517. doi: 10.1155/2019/6326517 30755787 PMC6348923

[59] Goldring SR. Alterations in periarticular bone and cross talk between subchondral bone and articular cartilage in osteoarthritis. Ther Adv Musculoskelet Dis. (2012) 4:249–58. doi: 10.1177/1759720X12437353 PMC3403248 22859924

[60] Chen Y Huang YC Yan CH Chiu KY Wei Q Zhao J. Abnormal subchondral bone remodeling and its association with articular cartilage degradation in knees of type 2 diabetes patients. Bone Res. (2017) 5:17034. doi: 10.1038/boneres.2017.34 29134132 PMC5674679

[61] Maruotti N Corrado A Cantatore FP. Osteoblast role in osteoarthritis pathogenesis. J Cell Physiol. (2017) 232:2957–63. doi: 10.1002/jcp.25969 PMC5575507 28425564

[62] Hilal G Martel-Pelletier J Pelletier JP Ranger P Lajeunesse D. Osteoblast-like cells from human subchondral osteoarthritic bone demonstrate an altered phenotype in *vitro*: possible role in subchondral bone sclerosis. Arthritis Rheumatol. (1998) 41:891–9. doi: 10.1002/1529-0131(199805)41:5<891::AID-ART17>3.0.CO;2-X 9588742

[63] Hopwood B Tsykin A Findlay DM Fazzalari NL. Microarray gene expression profiling of osteoarthritic bone suggests altered bone remodelling, WNT and transforming growth factor-beta/bone morphogenic protein signalling. Arthritis Res Ther. (2007) 9:R100. doi: 10.1186/ar2301 17900349 PMC2212557

[64] Abed E Bouvard B Martineau X Jouzeau JY Reboul P Lajeunesse D. Elevated hepatocyte growth factor levels in osteoarthritis osteoblasts contribute to their altered response to bone morphogenetic protein-2 and reduced mineralization capacity. Bone. (2015) 75:111–9. doi: 10.1016/j.bone.2015.02.001 25667190

[65] Mutabaruka MS Aoulad Aissa M Delalandre A Lavigne M Lajeunesse D. Local leptin production in osteoarthritis subchondral osteoblasts may be responsible for their abnormal phenotypic expression. Arthritis Res Ther. (2010) 12:R20. doi: 10.1186/ar2925 20141628 PMC2875652

[66] Wan Y Lv Y Li L Yin Z. 15-Lipoxygenase-1 in osteoblasts promotes TGF-β1 expression via inhibiting autophagy in human osteoarthritis. BioMed Pharmacother. (2020) 121:109548. doi: 10.1016/j.biopha.2019.109548 31704612

[67] Franco-Trepat E Alonso-Pérez A Guillán-Fresco M Jorge-Mora A Crespo-Golmar A López-Fagúndez M. Amitriptyline blocks innate immune responses mediated by toll-like receptor 4 and IL-1 receptor: Preclinical and clinical evidence in osteoarthritis and gout. Br J Pharmacol. (2022) 179:270–86. doi: 10.1111/bph.15707 PMC9300168 34643941

[68] Knights AJ Farrell EC Ellis OM Lammlin L Junginger LM Rzeczycki PM. Synovial fibroblasts assume distinct functional identities and secrete R-spondin 2 in osteoarthritis. Ann Rheum Dis. (2023) 82:272–82. doi: 10.1136/ard-2022-222773 PMC9972892 36175067

[69] Chen X Liu Y Wen Y Yu Q Liu J Zhao Y. A photothermal-triggered nitric oxide nanogenerator combined with siRNA for precise therapy of osteoarthritis by suppressing macrophage inflammation. Nanoscale. (2019) 11:6693–709. doi: 10.1039/C8NR10013F 30900717

[70] Ji Y Fang QY Wang SN Zhang ZW Hou ZJ Li JN. Lnc-RNA BLACAT1 regulates differentiation of bone marrow stromal stem cells by targeting miR-142–5p in osteoarthritis. Eur Rev Med Pharmacol Sci. (2020) 24:2893–901. doi: 10.26355/eurrev_202003_20653 32271407

[71] Fu K Robbins SR McDougall JJ. Osteoarthritis: the genesis of pain. Rheumatol Oxf Engl. (2018) 57:iv43–50. doi: 10.1093/rheumatology/kex419 29267879

[72] Ranasinghe P Addison ML Dear JW Webb DJ. Small interfering RNA: Discovery, pharmacology and clinical development-An introductory review. Br J Pharmacol. (2023) 180:2697–720. doi: 10.1111/bph.15972 36250252

[73] Bhavsar D Subramanian K Sethuraman S Krishnan UM. Translational siRNA therapeutics using liposomal carriers: prospects & challenges. Curr Gene Ther. (2012) 12:315–32. doi: 10.2174/156652312802083611 22856607

[74] Bartoszewski R Sikorski AF. Editorial focus: understanding off-target effects as the key to successful RNAi therapy. Cell Mol Biol Lett. (2019) 24:69. doi: 10.1186/s11658-019-0196-3 31867046 PMC6902517

[75] Janas MM Schlegel MK Harbison CE Yilmaz VO Jiang Y Parmar R. Selection of GalNAc-conjugated siRNAs with limited off-target-driven rat hepatotoxicity. Nat Commun. (2018) 9:723. doi: 10.1038/s41467-018-02989-4 29459660 PMC5818625

[76] Raja MAG Katas H Amjad MW. Design, mechanism, delivery and therapeutics of canonical and Dicer-substrate siRNA. Asian J Pharm Sci. (2019) 14:497–510. doi: 10.1016/j.ajps.2018.12.005 32104477 PMC7032099

[77] Hu B Zhong L Weng Y Peng L Huang Y Zhao Y. Therapeutic siRNA: state of the art. Signal Transduct Target Ther. (2020) 5:101. doi: 10.1038/s41392-020-0207-x 32561705 PMC7305320

[78] Yoshida T Delafontaine P. Mechanisms of IGF-1-mediated regulation of skeletal muscle hypertrophy and atrophy. Cells. (2020) 9:1970. doi: 10.3390/cells9091970 32858949 PMC7564605

[79] Øiestad BE Juhl CB Culvenor AG Berg B Thorlund JB. Knee extensor muscle weakness is a risk factor for the development of knee osteoarthritis: an updated systematic review and meta-analysis including 46 819 men and women. Br J Sports Med. (2022) 56:349–55. doi: 10.1136/bjsports-2021-104861 34916210

[80] Gomarasca M Banfi G Lombardi G. Myokines: The endocrine coupling of skeletal muscle and bone. Adv Clin Chem. (2020) 94:155–218. doi: 10.1016/bs.acc.2019.07.010 31952571

[81] Forcina L Miano C Scicchitano BM Musarò A. Signals from the niche: insights into the role of IGF-1 and IL-6 in modulating skeletal muscle fibrosis. Cells. (2019) 8:232. doi: 10.3390/cells8030232 30862132 PMC6468756

